# When study site contributes to outcomes in a multi-center randomized trial: a secondary analysis of decisional conflict in men with localized prostate cancer

**DOI:** 10.1186/s12955-014-0159-3

**Published:** 2014-10-25

**Authors:** Meghan L Underhill, Fangxin Hong, Donna L Berry

**Affiliations:** The Phyllis F. Cantor Center for Research in Nursing & Patient Care Services, Dana-Farber Cancer Institute, 450 Brookline Avenue, Boston, MA 02115 USA; Department of Biostatistics and Computational Biology, Dana-Farber Cancer Institute and Harvard School of Public Health, 450 Brookline Ave, CLSB-11017, Boston, Massachusetts 02215 USA

**Keywords:** Localized prostate cancer, Decisional conflict, Decision-making

## Abstract

**Purpose:**

Evaluate baseline factors that may explain the influence of study site on decisional conflict (DC) in men from the Personal Patient Profile: Prostate (P3P) randomized trial.

**Materials and methods:**

476 cases from 5 P3P sites were included. Participants completed baseline demographic assessments, 4 subscales of the DC scale at baseline (*uncertainty, informed, values clarity, and support*), the Expanded Prostate Cancer Index Composite (short form) and the State-Trait Anxiety Inventory. Site data regarding typical practices were collected. Linear regressions were used to model the relation between baseline DC scores and study site adjusting for the list of variables.

**Results:**

Baseline decisional *uncertainly* (p = 0.001) and *informed* (p = 0.03) subscales were significantly different across sites. Participant demographic and baseline measures were significantly different (p < 0.05) between sites except for trait anxiety. We identified participant level factors that explained study site differences at baseline for the decisional *uncertainty* and *values clarity* subscales: a preferred treatment choice at study entry, whether the study program was accessed at home vs. in clinic, number of doctors consulted pre-study, working status, state anxiety, information from the media or a health care provider, and perceived knowledge level. State anxiety was associated with higher DC across all subscales.

**Conclusions:**

Individual characteristics of men seeking consultation for LPC were associated with DC at baseline, not the site alone; anxiety contributed to higher conflict. These findings will inform future development and implementation of the P3P and other decision support interventions.

**Trial registration:**

NCT00692653.

## Background

In 2014, prostate cancer will account for 27% of new cancer cases and 10% of cancer related deaths in men [1]. Over 90% will be diagnosed with localized prostate cancer (LPC) [1]. There are a variety of care and treatment “options” for men diagnosed with LPC including surgical treatment, radiation treatment, or active surveillance. There is little medical evidence to support the “best” option for most men diagnosed with LPC and therefore men often are asked to contribute to the care or treatment decision. These decisions are complex and are made based on a variety of personal and social economic factors, as well as the side effect profile of each approach [2-6].

The Personal Patient Profile: Prostate (P3P) is a Web-based intervention providing tailored, values-based education and communication coaching to men making decisions about management of LPC [7]. The P3P was tested in a multi-site randomized control trial (RCT) from 2007–2009 comparing standard patient education plus P3P to standard patient education alone [8]. The 6 study sites in 4 U.S. cities were the Seattle Prostate Institute (SPI) in Seattle, Washington; the University of Washington (UW)/Seattle Cancer Care Alliance (SCCA) in Seattle, Washington; Fox Chase Cancer Center (FCCC) radiation oncology clinic in Philadelphia, Pennsylvania; and Veterans Affairs Hospitals (VA) in Seattle, Washington; San Antonio, Texas; and Augusta, Georgia.

The main outcome of the trial was decisional conflict over 6 months, measured by the validated Decisional Conflict (DC) scale [9]. A total of 494 eligible cases participated in the original study. A detailed description of the study sample and procedures has been previously reported [8]. P3P was found to significantly reduce DC related to making LPC treatment decisions over 6 months, adjusting for participant personal characteristics and baseline measures [8].

In the primary multivariable analysis, even after controlling for participant personal characteristics and other study measures, the study site at which participants received consultation remained a significant predictor of DC [8,10]. The purpose of this analysis was to explore further the factors that could potentially explain the influence of study site on DC, focusing on site characteristics and pre-intervention variables measured at baseline prior to entry into the study and receiving consultation at the study site.

## Methods

### Participants

The study sites were described in detail elsewhere [8] and summarized in Table 1. One participating site (SPI) enrolled only 18 participants, compared to 25 or more at all other sites, and was therefore excluded due to small sample size. 476 cases from 5 sites were included in this analysis. Institutional Review Board approval was obtained at each site for the original trial, with the University of Washington/Fred Hutchinson Cancer Consortium as the lead IRB site, and all participants had completed written informed consent.

**Table 1:** **Description of baseline participant characteristics and study measures across study sites**

	**Site**										**p-value**
	**Augusta**		**Philadelphia**		**San Antonio**		**Seattle UW-SCCA**		**Seattle Puget Sound-VA**		
	**(n=91)**		**(n=88)**		**(n=25)**		**(n=225)**		**(n=47)**		
	**Median (range)**		**Median (range)**		**Median (range)**		**Median (range)**		**Median (range)**		
Age	62 (45–78)		66	(43–79)	62	(52–77)	62	(40–86)	63	(52–78)	0.03
	**N**	**(%)**	**N**	**(%)**	**N**	**(%)**	**N**	**(%)**	**N**	**(%)**	
College education or higher	23	25.3	51	58.0	6	24.0	168	74.7	18	38.3	<.0001
Caucasian	39	42.9	84	95.5	12	48	214	95.1	40	85.1	<.0001
Income 35K or less	54	59.3	7	8.0	8	32.0	16	7.1	24	51.1	<.0001
Married/partnered	56	61.5	72	81.8	20	80.0	188	83.6	27	57.4	<.0001
Working (yes)	40	44.0	55	62.5	11	44.0	137	60.9	19	40.4	.005
Program access location (at Clinic)	82	90.1	9	10.2	9	36.0	37	16.4	13	27.7	<.0001
Having a treatment choice at baseline	51	56.0	41	46.6	15	60.0	96	42.7	33	70.2	0.003
Number of doctors consulted											<.0001
0	58	63.7	9	10.2	9	36	52	23.1	18	38.3	
1	27	29.7	24	27.3	11	44.0	83	36.9	13	27.7	
> 1	6	6.6	55	62.5	5	20.0	90	40.0	16	34.0	
**Perceived knowledge**	34	37.4	67	76.1	7	28.0	147	65.3	23	48.9	<.0001
Fair/A lot											
Some	26	28.6	14	15.9	9	36.0	57	25.3	8	17.0	
None/little	31	34.1	7	8.0	9	36.0	21	9.3	16	34.0	
**Information sources (yes)**											
Self	16	17.6	45	51.1	6	24.0	102	45.3	16	34.0	<.0001
Health care provider	79	86.8	58	65.9	22	88.0	166	73.8	43	91.5	0.0005
Media	49	53.8	77	87.5	16	64.0	192	85.3	23	48.9	<.0001
Other people	58	63.7	72	81.8	15	60.0	178	79.1	31	66.0	0.005
**STAI**	**M**	**SD**	**M**	**SD**	**M**	**SD**	**M**	**SD**	**M**	**SD**	
State anxiety	35.7	13.4	42.6	15.0	40.3	16.3	40.6	12.2	39.0	11.9	0.009
Trait anxiety	34.2	11.4	33.7	11.0	35.6	12.9	32.5	8.8	36.0	11.2	0.16
**EPIC-SF questionnaire**											
Urinary irritative	87.5	16.5	93.8	11.1	87.5	18.2	87.5	15.8	87.5	14.8	0.02
Urinary incontinence	100	16.2	100	10.4	91.8	16.8	100	12.8	100	13.9	0.02
Bowel symptoms	100	13.4	100	9.7	95.8	13.2	100	9.8	100	12.0	0.04
Sexual symptoms	61.8	32.6	72.3	30.6	30.5	29.4	78.5	26.9	66.7	28.5	<.0001
Hormonal symptoms	90	14.8	95	12.2	85	20.7	95	11.0	90	10.6	<.0001

Note: M = Mean, SD = standard deviation STAI = State Trait Anxiety Inventory (scores range from 20–80 with higher scores indicating more anxiety), EPIC-SF = Expanded Prostate Index Composite-Short Form (scores range from 0–100 with higher scores indicating better HRQOL); One-way ANOVA and Chi-square test were used for comparing means and proportions across sites.

### Measures

Participants in the original RCT self-reported personal characteristics, concerns and preferences, all previously documented [2] as important to prostate cancer treatment decision making. Additional self-reported variables included the number of doctors consulted about prostate cancer treatment prior to study enrollment and level of perceived knowledge about prostate cancer and its treatment. Participants also were asked *do you think you know which treatment you want* (yes/no) and *how many weeks has it been since your prostate biopsy.* One item in the original trial asked the participant to select what type of prostate cancer information sources that had been used prior to enrollment. This variable was re-coded into four dichotomous (yes/no) variables related to information source: self (*books, pamphlets that I got myself*), health care provider (*books, pamphlets that my health care providers gave me)*, media (*magazines, newspapers, Internet, television/videos*), and other people (*family members, others*).

The DC questionnaire [9] was completed at baseline. Because the eligible participants all were scheduled for a consult about a care or treatment decision and had not made a final decision, only the first 4 DC subscales were presented at baseline and included in this analysis: uncertainty, informed, values clarity, and support (Table 2). Item score responses ranged from 0 (agree) to 4 (strongly disagree) with higher scores representing more conflict. Subscale scores were transformed 0 (no DC) to 100 (extremely high DC) [11]. The Expanded Prostate Cancer Index Composite (short form) (EPIC) was reported, measuring prostate specific, health related quality of life (HRQOL) [12]. Higher scores indicate better HRQOL in the domains of irritative urinary symptoms, urinary incontinence, hormone-related side effects, and bowel and sexual function. Item scores were transformed to a 0 to 100 scale and the average scores within each subscale were taken to create subscale scores [13]. The Spielberger State-Trait Anxiety Inventory (STAI) was used to assess baseline anxiety. Item scores for STAI are summated for each subscale (state and trait) and scores range from 20–80 with higher scores indicating more anxiety [14].

**Table 2:** **Four subscales and items of the decisional conflict scale used at baseline** [8,9]

**Uncertainty**	• I am clear about the best choice for me.
Higher score = greater uncertainty	• I feel sure about what to choose.
	• This decision is easy for me to make.
**Informed**	• I know which options are available to me.
Higher score = less informed	• I know the benefits of each option.
	• I know the risks and side effects of each option.
**Values Clarity**	• I am clear about which benefits matter most to me.
Higher score = lack of clarity about personal values	• I am clear about which risks and side effects matter most.
	• I am clear about which is more important to me (the benefits or the risks and side effects).
**Support**	• I have enough support from others to make a choice.
Higher score = lack of support	• I am choosing without pressure from others.
	• I have enough advice to make a choice.

Responses for each item range from 0 (strongly agree) to 4 (strongly disagree); adapted from [7].

Additional data about the study site clinical processes were collected after the original trial and prior to the secondary analysis during interviews with study site investigators. The investigators were asked to “*Describe the typical practices and process a man with localized prostate cancer would follow from detection and biopsy to making a care decision*’. Investigators were prompted to report specific information about the clinical process in general regarding number of consultations, length of time between biopsy and disclosure of results, and/or treatment decision making, type of specialists at the site, length of time between consult visit, method of disclosing biopsy results, and if educational material was given to patients. Based on the responses, 5 categorical variables were created.

### Statistical methods

Baseline patient characteristics and study measures were compared among sites. One-way ANOVA and Chi-square test were used for means and proportions, respectively. All analyses were conducted using SAS (version 9.2).

For each of the four DC subscales, linear regression was used to model the relation between baseline DC scores and study site adjusting for personal characteristics (or factors) and the site level variables. We explored whether study site remained a significant variable associated with baseline DC scores after adjusting for additional baseline factors. First, univariate analysis was performed between each factor. Factors that were potentially associated with DC score, with significance levels (p-value) less than 0.2 were included in the multivariable model. Backward variable selection was used to identify significant predictors, where a variable was statistically significant if p-value ≤0.05 and kept in the model if p-value ≤0.2. Possible two-way interactions among remaining predictors were examined. Site level variables exhibited multi-collinearity with study site and were not included in the multivariable model.

## Results

### Overview

The participant characteristics, including demographic, reports of knowledge level, information source, anxiety and symptom, were significantly different across sites (Table 1). The men enrolled in Philadelphia at Fox Chase and at the Seattle UW/SCCA were predominately college-educated, Caucasian, working and had accessed the baseline P3P measures on a personal computer or tablet. In contrast, men enrolled in the Veterans Administration hospital sites (Augusta, San Antonio and Seattle) were predominately high school educated, not working, had accessed the baseline measures in the clinic on a study computer and about half were of minority race or ethnicity. Further, the majority of men in Philadelphia and Seattle UW/SCCA had already consulted with other physicians since the time of biopsy, had retrieved prostate cancer information themselves and from media, and reported a higher level of perceived prostate cancer knowledge.

**Figure 1:**
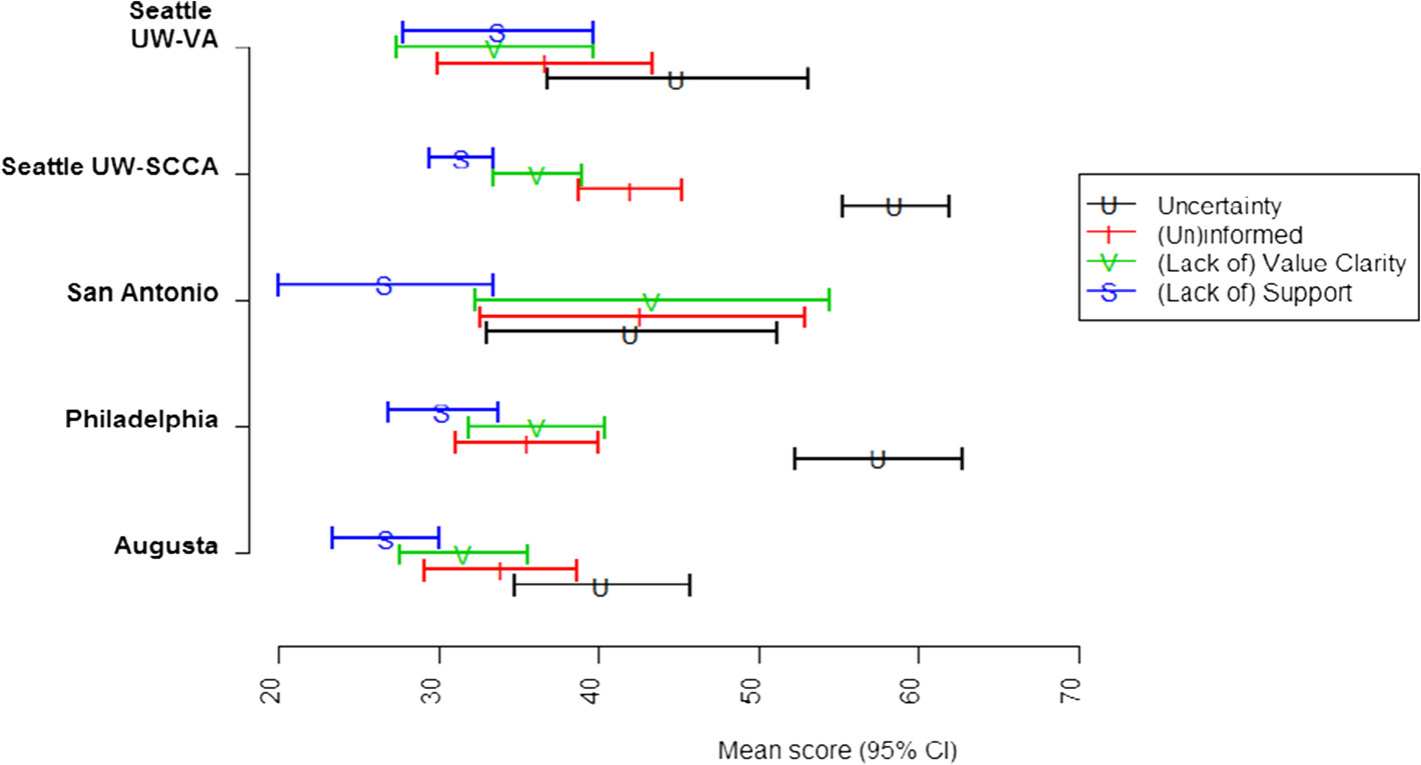
**Mean scores together with 95% confidence interval (CI) describing the variation in the four DCS subscales measured at baseline across sites.** *Note: Higher mean scores indicate more Decisional Conflict; ANOVA testing mean difference across sites: Uncertainty subscale p<0.001; Informed subscale p = 0.03; values clarity subscale p = 0.12; support subscale p = 0.07.

Figure 1 illustrates the mean scores with 95% confidence intervals (CI) for the four DC subscales measured at baseline; baseline decisional uncertainly (p < 0.001) and informed (p = 0.03) subscale scores were significantly different across sites. The effect of the baseline personal characteristics and reports of knowledge level, information sources, anxiety and symptoms on DC subscale scores were estimated in both univariate (Table 3) and multivariable (Table 4) analyses.

**Table 3:** **The influence of personal factors on decisional conflict subscale scores-results from the univariate analysis**

**Variable**	**Decisional uncertainty**		**Informed subscale**		**Values clarity subscale**		**Support subscale**	
	**Estimate**	**p-value**	**Estimate**	**p-value**	**Estimate**	**p-value**	**Estimate**	**p-value**
*Study center		<.0001		0.03		0.13		0.07
Augusta	−4.67		−2.72		−1.99		−7.00	
Philadelphia	12.62		−1.10		2.60		−3.47	
San Antonio	−2.86		6.05		9.82		−7.02	
Seattle UW-SCCA	13.66		5.37		2.61		−2.32	
Age	−0.24	0.17		0.87		0.62		0.81
Treatment preference at baseline (no vs. yes)	27.3	<.0001	16.6	<.0001	14.32	<.0001	8.40	<.0001
Education (college no vs. yes)	−6.62	0.008		0.54		0.22		0.90
Income (>35K vs. <35k)	10.8	0.0002	3.64	0.16		0.63		0.60
Caucasian (no vs. yes)	−14.5	<.0001	−4.39	0.12		0.52	−3.22	0.10
P3P access (Clinic vs. Not Clinic)	−13.8	<.0001	−3.76	0.11	−5.72	0.007	−2.40	0.14
Married/partnered (no vs. yes)		0.93		0.28		0.58	4.51	0.01
Number of doctors consulted		0.0004		<.0001		0.003		0.002
0 vs. >1			13.55		7.94		5.64	
1 vs. >1			5.54		2.10		−0.05	
Working status (No vs. yes)	−6.05	0.02	−3.08	0.16		0.27		0.86
**Perceived knowledge**		0.38		<.0001		<.0001		<.0001
Fair/A lot vs. some			−19.52		−11.72		−3.87	
None/little vs. some			3.76		6.88		7.73	
**STAI								
State anxiety	0.75	<.0001	0.50	<.0001	0.41	<.0001	0.44	<.0001
Trait anxiety	0.31	0.01	0.35	0.001	0.35	0.0002	0.43	<.0001
***EPIC scale								
Urinary irritative symptoms	−0.1	0.2	−0.1	0.18		0.17		0.61
Urinary incontinence symptoms		1.0		0.82		0.81		0.45
Bowel symptoms	0.1	0.39		0.62		0.65		0.92
Sexual symptoms	0.06	0.16	−0.08	0.02	−0.06	0.07		0.90
Hormonal symptoms	−0.03	0.72	−0.2	0.02	−0.15	0.05	−0.16	0.005
**Information Sources**								
Self (no vs. yes)	−8.16	0.001	7.32	0.001	4.89	0.01		0.84
Health care provider (no vs. yes)	4.08	0.17	7.27	0.005	7.10	0.002	4.22	0.02
Media (no vs. yes)	−13.98	<.0001		0.56		0.57		0.81
Other people (no vs. yes)		0.22		0.25		0.70		0.59

Note: Higher subscale scores indicate more decisional conflict; *Seattle/University of Washington VA is the reference group; **STAI = State Trait Anxiety Inventory; ***EPIC = Expanded Prostate Index Composite-Short Form.

**Table 4:** **Factors that explain study site influence on decisional conflict-results from the multivariable analysis**

**Variable**	**Decisional uncertainty**		**Informed subscale**		**Values clarity subscale**		**Support subscale**	
	**Est.**	**p-value**	**Est.**	**p-value**	**Est.**	**p-value**	**Est.**	**P-value**
*Study center		0.47		0.003		0.52		0.002
Augusta			−5.58				−6.69	0.01
Philadelphia			0.96				−0.19	0.95
San Antonio			0.73				−7.56	0.04
Seattle UW-SCCA			5.83				0.51	0.84
Treatment preference at baseline (no vs. yes)	24.5	<.0001	10.92	<.0001	10.44	<.0001	4.87	0.0005
Caucasian (No vs. Yes)	−4.30	0.16	−4.23	0.13				
Intervention Access (Clinic vs. Not Clinic)	−6.33	0.02			−6.17	0.01		
Married/Partnered (no vs. yes)							2.95	0.07
Number of doctors		0.006		0.002		0.14		0.05
0 vs. >1	−5.68	0.01	9.18		4.9		4.51	0.01
1 vs. >1	−7.52	0.001	5.29		2.0		1.17	0.47
Working status (no vs. yes)	−2.85	0.15	−4.25	0.03				
**Perceived knowledge**				<.0001		<.0001		<.0001
Fair/A lot vs. some			−16.55		−10.55		−7.00	<.0001
None/little vs. some			3.03		5.41		2.45	0.26
**STAI								
State	0.53	<.0001	0.39	<.0001	0.27	<.0001	0.36	<.0001
Trait			−0.15	0.19				
***EPIC scale								
Sexual symptoms			−0.07	0.04				
**Information source**								
Self (no vs. yes)			2.53	0.21				
Health care provider (no vs. yes)			3.09	0.17	3.94	0.07		
Media (no vs. yes)	−7.74	0.002						

Note: Higher estimates indicate more decisional conflict; *Seattle/Puget Sound Veteran’s Administration Hospital is the reference group; **STAI = State Trait Anxiety Inventory; ***EPIC = Expanded Prostate Index Composite-Short Form.

### Additional site level variables

Univariate analyses revealed sites that typically disclosed the diagnosis in person in the clinic, provided more than one in-person visit, took greater than one month between biopsy to treatment decision, and did not conduct follow-up by telephone had enrolled participants with significantly less decisional conflict related to uncertainty. Sites that provided educational information (handouts, books), enrolled participants that reported significantly more decisional conflict related to uncertainty. Sites that disclosed the diagnosis in person enrolled participants that reported higher scores on the informed subscale and those that had less than 1 month from biopsy to treatment decision and gave educational handouts had participants that reported lower DC on the informed subscale (data reported in Table 5). These site level variables were collinear with the overall study site variable and therefore not included in subsequent multivariable analysis.

**Table 5:** **Univariate analysis of the relationship between decisional conflict and the additional site level variables collected**

**Site Variable**	**Decisional uncertainty subscale**		**Informed subscale**		**Values clarity subscale**		**Support subscale**	
	**Est.**	**p-value**	**Est.**	**p-value**	**Est.**	**P-value**	**Est.**	**P-value**
Number of in person visits (>1 vs. . 1)	−14.5	0.008	2.97	0.55	7.55	0.09	−4.72	0.16
Diagnosis disclosure method		.0001		0.09		0.96		0.85
In person vs. telephone	−14.7	<.0001	−3.27	0.31	0.85	0.77	−0.12	0.96
N/A vs. telephone	−1.0	0.75	−6.47	0.03	−0.11	1.0	−1.16	0.57
Time from Biopsy to treatment decision		0.0001		0.09		0.96		0.85
<1 mo vs. N/A	1.0	0.75	6.47	0.03	0.01	1.0	1.16	0.57
> 1 mo vs. N/A	−13.6	0.001	3.21	0.40	0.86	0.10	1.04	0.69
Educational material given (no vs. yes)	7.16	0.008	5.03	0.04	−0.37	0.87	0.69	0.68
Telephone follow-up given (no vs. yes)	−14.5	0.008	2.97	0.55	7.55	0.09	−4.72	0.16

Note: Higher estimates indicate more decisional conflict.

### Decisional uncertainty subscale

The baseline mean scores on the decisional uncertainly subscale were significantly higher at the Philadelphia Fox Chase radiation oncology site and the Seattle UW/SCCA site (Figure 1). In univariate analysis (Table 3), not having a treatment preference at baseline, Caucasian race, college education, income greater than $35,000, accessing the P3P intervention at home, working, having higher STAI scores, and seeking information independently or from the media were associated with significantly higher decisional uncertainty scores. Seeing more than one doctor also was associated with higher decisional uncertainty scores.

When participant level variables were entered in the multivariable model, site was no longer a statistically significant (p = 0.47) predictor of DC (Table 4). Lower decisional uncertainty was significantly associated with a treatment choice at study entry and accessing the P3P program in the clinic. Higher uncertainty was significantly associated with having seen more than one doctor, higher state anxiety, and obtaining information from the media.

### Informed subscale

Variables that were associated in univariate analysis (Table 3) with lower scores on the informed subscale were not having a treatment decision at study entry, high state anxiety, accessing P3P at home, having less knowledge, and no pre-study sexual or hormonal symptoms. Higher scores on the informed subscale were significantly associated with seeking information independently or from a health care provider.

Though all potential baseline variables trending towards significance in the univariate model were included in the multivariable analysis (Table 4), study site remained a significant factor (p = 0.003) for the informed subscale. The aspect of DC related to being informed was significantly lower at the Augusta site, meaning participants at this site reported a perception of being more informed. Participants who reported having a treatment preference at study entry, seeing more than one pre-study doctor, having sexual function issues, and higher prostate cancer knowledge reported being more informed. Not working and higher state anxiety scores were significantly associated with a report of being less informed.

### Values clarity subscale

Not having a decision at baseline, accessing P3P at home, reporting less knowledge, higher STAI, and no hormonal symptoms were significantly associated with having higher values clarity scores, indicating more conflict in univariate analyses (Table 3). Seeking information independently or from the health care provider was associated with lower scores indicating more values clarity. Multivariable analyses (Table 4) indicated that study site was not significantly (p = 0.52) associated with values clarity. State anxiety was significantly associated with a lack of values clarity (higher subscale score). Lower values clarity subscale scores, indicating less conflict, were associated with having a treatment preference at study entry, accessing the study program at the study site and reporting a higher level of prostate cancer knowledge.

### Support subscale

In univariate analyses (Table 3), having seen more than one doctor pre-study, having a treatment decision at study entry, reporting more knowledge, and baseline hormonal symptoms were associated with lower DC related to support needed to make a decision. Higher state and trait anxiety, being single, fewer pre-study consults, were significantly associated with conflict related to less support.

Study site remained a significant factor (p = 0.002) after conducting the multivariable analysis (Table 4). Participants enrolled at the Augusta and San Antonio VA hospitals reported significantly less DC related to support at baseline. Factors associated with lower support subscale scores, and therefore having enough support for the decision thus far, were: having a treatment preference at study entry, having seen more than one doctor pre-study, and reporting a higher level of prostate cancer knowledge. Higher state anxiety scores were significantly associated with higher DC support scores.

## Discussion

In the multisite P3P trial, the study site where participants received consultation served significantly different patient populations and study site was a significant factor associated with the main outcome of DC at 6 months. The current analysis explained what factors played a role in the significance of study site for two of the four DC subscales, uncertainty and values clarity. For the remaining two subscales included in this analysis, informed and support, study site remained significant. Potentially unmeasured variables play a role in why study site is important for these subscales and future trials will measure study site specific variables as well as participant factors.

The primary finding from our secondary analysis documents that the characteristics of men who sought treatment at the sites were explanatory of baseline DC measures. Disproportionate numbers of men with certain characteristics were consulted at the various study sites. The results highlight how men from diverse backgrounds engage in the complexity of decision making for LPC treatment. Race was implicated in univarate analysis of uncertainty, but did not predict DC once entered into the multivariable model. For those men with higher socioeconomic status, such as those at the Philadelphia Fox Chase radiation oncology site and the Seattle UW/SCCA, with access to information and health care resources, who sought information independently, and accessed Web-based information and multiple doctors to discuss treatment options (by default at the radiation site), DC related to uncertainty was higher. These men may have known the complexity of the LPC treatment decision at study entry, and therefore experienced more conflict. In contrast, men with lower socioeconomic status, such as those from the VA hospital sites, who did not access information outside of the clinic and did not seek information independently may have been less aware of the complexities and implications of the multiple treatment options available for LPC, and therefore experienced less conflict at study entry.

The significant influence of scores from the EPIC hormonal symptom domain was an interesting finding in the univariate analysis for three of four DC scales, though not sustained in the multivariable model. Men at the San Antonio VA had significantly worse scores in this domain. Certainly, at diagnosis these men had not been exposed to any androgen suppressive therapies. Two of the items within this domain inquire about depression and energy; this may help explain the relationship between the EPIC hormonal domain scores and DC.

Having a preferred treatment choice at study entry and having higher perceived knowledge were important factors that contributed to lower DC across subscales. It is important to recognize that having perceptions of a preferred treatment decision and having high perceived knowledge prior to having had LPC treatment related consultation does not equate with actual knowledge about LPC treatment options. A descriptive study [15] evaluating prostate cancer related knowledge in 109 men with and without a prostate cancer diagnosis from low income settings, found low to moderate levels of prostate cancer knowledge and comprehension in the sample. The findings that of the age appropriate men, fewer than half knew the various prostate cancer treatment options and less than a fourth knew the potential side effects of treatment, may help place our findings in context. Men with low socioeconomic status may perceive themselves as having adequate knowledge but still require more informational support related to prostate cancer and treatment. In a recent publication, Kaplan et al. [16] reported baseline factors that predicted DC scores in men with LPC from a VA clinic. They reported that lower prostate cancer knowledge was associated with higher DC and uncertainty at baseline. In the P3P RCT, men at sites, such as the Seattle VA site, that had the lowest baseline DC scores, indicating men were the least conflicted, actually had the largest difference between control and intervention groups with regard to overall DC scores six months from enrollment [8]. Our secondary analysis helps us understand that participants who were not as prepared at baseline may have been less aware of the complexity of the decision and, the educational and coaching component of the P3P intervention benefited these men over time as they engaged in the complex task of a shared decision.

If a man had high state, or situational, anxiety at study entry, he was more likely to have higher DC across all subscales. State anxiety is a modifiable factor that could be addressed by clinical teams providing consultation for LPC treatment. Davison and colleagues [17] reported significantly lower state anxiety in men with newly diagnosed prostate cancer six weeks after randomization to an informational intervention. In 2012, the American College of Surgeons Commission on Cancer proposed a 2015 mandate for cancer care settings that the management of distress, that includes anxiety, should be a component of all patient care [18].

Limitations to our findings are important to identify. Study site process variables were measured by investigator recall and the current analysis may not have included all necessary site information, potentially missing important contributions that unknown study site variables may have made to the results.

### Clinical implications

Clinicians who consult with men regarding management of LPC may use these findings to better support men and decrease DC. Anxiety could be assessed at baseline to help clinicians understand which patients are likely to feel most conflicted when making a treatment decision and then target support to specific patient needs. Clinical centers which serve a high proportion of men with no access to the Internet and who are typically not consulting with multiple clinicians may want to assure (not assume) that men have a full understanding of prostate cancer and options for treating or monitoring the condition.

## Conclusions

Individual characteristics of men seeking consultation for localized prostate cancer were associated with DC at baseline and men with these characteristics were enrolled disproportionately at the various sites. While the original impact of the P3P intervention was positive despite these site differences, we now understand more of the influential baseline factors, notably information access and perceptions of knowledge about prostate cancer options. The modifiable factor of anxiety was identified as contributing to higher conflict at baseline. These finding will inform future development and implementation of the P3P and other decision support interventions.

## Abbreviations

P3P: Personal patient profile: Prostate
LPC: Localized prostate cancer
DC: Decisional conflict
STAI: State trait anxiety index

## References

[1] American Cancer Society (2014). Cancer Facts and Figures 2014.

[2] Berry DL, Ellis WJ, Russell KJ, Blasko JC, Bush N, Blumenstein B, Lange PH (2006). Factors that predict treatment choice and satisfaction with the decision in men with localized prostate cancer. Clin Genitourin Cancer.

[3] Sommers BD, Beard CJ, D’Amico AV, Kaplan I, Richie JP, Zeckhauser RJ (2008). Predictors of patient preferences and treatment choices for localized prostate cancer. Cancer.

[4] Davison BJ, Breckon E (2012). Factors influencing treatment decision making and information preferences of prostate cancer patients on active surveillance. Patient Educ Couns.

[5] Holmboe ES, Concato J (2000). Treatment decisions for localized prostate cancer. J Gen Intern Med.

[6] Steginga SK, Occhipinti S, Gardiner RA, Yaxley J, Heathcote P (2002). Making decisions about treatment for localized prostate cancer. BJU Int.

[7] Berry DL, Halpenny B, Wolpin S, Davison BJ, Ellis WJ, Lober WB, McReynolds J, Wulff J (2010). Development and evaluation of the personal patient profile-prostate (P3P), a Web-based decision support system for men newly diagnosed with localized prostate cancer. J Med Internet Res.

[8] Berry D, Halpenny B, Hong F, Wolpin S, Lober W, Russell K, Ellis W, Govindarajulu U, Bosco J, Davison B: **The Personal Patient Profile-Prostate decision support for men with localized prostate cancer: a multi-center randomized trial.** *Urol Oncol* 2011**:**1012 10.1016/j.urolonc.2011.10.004 PMC3349002 22153756

[9] O’Connor AM (1995). Validation of a decisional conflict scale. Med Decis Mak.

[10] Berry D, Hong F, Halpenny B, Wolpin S, Lober W, Russell K, Ellis W, Davison BJ, Barsevick A, Yang C (2011). 1001 decisional conflict varies by institution for men making treatment decisions for localized prostate cancer. J Urol.

[11] O’Connor, A: **User Manual-Decisional Conflict Scale 1993.** See [https://decisionaid.ohri.ca/eval.html#DecisionalConflictScale] (last checked 27 October 2009).

[12] Szymanski KM, Wei JT, Dunn RL, Sanda MG (2010). Development and validation of an abbreviated version of the expanded prostate cancer index composite instrument for measuring health-related quality of life among prostate cancer survivors. Urology.

[13] **Scoring Instructions for the Expanded Prostate cancer Index Composite Short Form (EPIC-26)** [http://www.med.umich.edu/urology/research/EPIC/EPIC-26-Scoring-1.2007.pdf]

[14] Spielberger CD (1983). State Trait Anxiety Inventory for Adults: Sampler Set: Manual, Test, Scoring Key; [form Y]; STAIS-AD.

[15] Wang DS, Jani AB, Tai CG, Sesay M, Lee DK, Goodman M, Echt KV, Kilbridge KE, Master VA (2013). Severe lack of comprehension of common prostate health terms among low-income inner-city men. Cancer.

[16] Kaplan AL, Crespi CM, Saucedo JD, Connor SE, Litwin MS, Saigal CS (2014). Decisional conflict in economically disadvantaged men with newly diagnosed prostate cancer: baseline results from a shared decision-making trial. Cancer.

[17] Davison BJ, Degner LF (1997). Empowerment of men newly diagnosed with prostate cancer. Cancer Nurs.

[18] **Cancer Program Standards 2010: Ensuring Patient-Centered Care** [http://www.facs.org/cancer/coc/programstandards2012.pdf]

